# New insights into the fungal community from the raw genomic sequence data of fig wasp *Ceratosolen solmsi*

**DOI:** 10.1186/s12866-015-0370-3

**Published:** 2015-02-12

**Authors:** Li-Hua Niu, Xiu-Feng Song, Shun-Min He, Peng Zhang, Ning-Xin Wang, Yi Li, Da-Wei Huang

**Affiliations:** College of Plant Protection, Shandong Agricultural University, Tai’an, China; Key Laboratory of Zoological Systematics and Evolution, Institute of Zoology, Chinese Academy of Sciences, Beijing, China; College of Environment, Hohai University, Nanjing, China

**Keywords:** Fungal community, Unmapped raw data, Fig wasp, Fungal reference datasets

## Abstract

**Background:**

To date, biologists have discovered a large amount of valuable information from assembled genomes, but the abundant microbial data that is hidden in the raw genomic sequence data of plants and animals is usually ignored. In this study, the richness and composition of fungal community were determined in the raw genomic sequence data of *Ceratosolen solmsi* (RGSD-CS).

**Results:**

To avoid the interference from sequences of *C. solmsi*, the unmapped raw data (about 17.1%) was obtained by excluding the assembled genome of *C. solmsi* from RGSD-CS. Comparing two fungal reference datasets, internal transcribed spacer (ITS) and large ribosomal subunit (LSU) of rRNA, the ITS dataset discovered a more diverse fungal community and was therefore selected as the reference dataset for evaluating the fungal community based on the unmapped raw data. The threshold of 95% sequence identity revealed many more matched fungal reads and fungal richness in the unmapped raw data than those by identities above 95%. Based on the threshold of 95% sequence identity, the fungal community of RGSD-CS was primarily composed of Saccharomycetes (88.4%) and two other classes (Agaricomycetes and Sordariomycetes, 8.3% in total). Compared with the fungal community of other reported fig wasps, Agaricomycetes and Eurotiomycetes were found to be unique to *C. solmsi*. In addition, the ratio of total fungal reads to RGSD-CS was estimated to be at least 4.8 × 10^−3^, which indicated that a large amount of fungal data was contained in RGSD-CS. However, rarefaction measure indicated that a deeper sequencing coverage with RGSD-CS was required to discover the entire fungal community of *C. solmsi*.

**Conclusion:**

This study investigated the richness and composition of fungal community in RGSD-CS and provided new insights into the efficient study of microbial diversity using raw genomic sequence data.

**Electronic supplementary material:**

The online version of this article (doi:10.1186/s12866-015-0370-3) contains supplementary material, which is available to authorized users.

## Background

Microbes are ubiquitous in most plant and animal organs and contribute various functions that affect the survival and metabolism of hosts. In recent years, many scientists focused on bacterial communities that are related to insects and identified kinds of important roles of bacteria in insects, including their roles in reproduction, immunity, and nutrition of their insect hosts [1-3]. Besides bacteria, fungi also serve important roles in their insect hosts [4-6]. However, very few studies have addressed the fungal communities of insects, which have impeded further exploration of the functional relationship between fungal communities and their insect hosts.

Cultivation of fungi in the lab is the most traditional method for further analysis, but this approach limited the quick finding of amount of uncultured fungal species [7]. Consequently, culture-independent methods have been commonly applied in more recent studies. These methods, including denaturing gradient gel electrophoresis (DGGE), temperature gradient gel electrophoresis (TGGE), terminal restriction fragment length polymorphism (T-RFLP), and clone libraries, which are based on a barcoding fragment of a conserved gene, can be used to quickly and cheaply determine the main components of fungal communities [8,9]. However, many low-abundance or rare fungal components are not revealed using these methods because of the small amounts of sequence data [10,11]. High-throughput sequencing with metabarcoding of DNA has minimized these issues by providing a large amount of sequence data. Although this valuable method has been used by scientists to discover multiple important findings regarding the relationships between microbes and their hosts [12-15], the targeted sequencing for large amounts of fungal barcoding data is relatively expensive.

In parallel with the technological advancement of high-throughput sequencing, there has been an increase in the genomic sequencing of organisms. By 2014, at least 134 genomes of representative insect species have already been assembled and released, and hundreds of other insect genomes are being sequenced or prepared for sequencing. However, an increasing amount of sequence contaminations from microbes were also discovered in some of these assembled genomes [16-18]. It is easy for us to speculate that the large amount of raw genomic sequence data of insects (approximately hundreds of gigabytes per insect) should also contain a large amount of microbial sequence data in addition to the targeted genomic sequences of insects. However, there has been no direct report regarding microbial information in the raw genomic sequence data of insects.

The fig-fig wasp system is a classic model for the study of coevolution between plants and insects [19,20]. The small and enclosed fig syconium exerts strong pressure on fig wasps and relevant microbes. Fig wasps have experienced various types of morphological evolution, such as males evolving the absence of wings, antennae, and eyes [20,21]. The genome of *Ceratosolen solmsi* also shows marked reductions of gene families that are involved in chemosensory-related, detoxification, and cuticular protein genes [22]. The bacterial community of fig wasps is structured by the hosts’ ecological niches but not the fig wasp phylogeny [23]. Some *C. solmsi* bacteria are also revealed to be related to certain nutrients, such as arginine [22]. However, little is known regarding the fungal community of fig wasps. Some studies using culture-dependent methods reported that only 1 fungal species existed in fig wasps [24]. The only systematic investigation on the fungal community of the fig-fig wasp system was carried out by Sanger sequencing with 313 internal transcribed spacer (ITS) sequences which corresponded to 27 operational taxonomic units (OTUs, based on 95% sequence identity) [25].

In order to improve our understanding of the fungal community of fig wasps and to determine the amount fungal sequence data present in the raw genomic sequence data of *Ceratosolen solmsi* (abbr. RGSD-CS), we first investigated the fungal community in RGSD-CS by pair-wise alignment between the raw reads and two reference datasets of fungi. We then calculated the proportion of fungal data in RGSD-CS by conducting similar alignments between the raw reads and 773 released fungal genomes.

## Results

### Proper parameters for the screening of fungal sequences in RGSD-CS

Prior to screening fungal reads in RGSD-CS, the unmapped raw data was obtained from RGSD-CS, by excluding the assembled genome of *C. solmsi* which matched RGSD-CS with 100% similarity. The unmapped raw data contained 96,749,007 reads (http://www.regulatoryrna.org/pub/figwasp/fungal/) and accounted for 17.05% of RGSD-CS.

Although a uniform threshold of fungal sequence identity for intra- and inter- genus or higher taxon was rarely defined, 95% identity has been commonly employed as the threshold for the intra- and inter- species discrimination of fungi [26,27]. Thus, a set of sequences with identities above or equal to 95%, were compared to investigate the fungal community in RGSD-CS.

**Figure 1:**
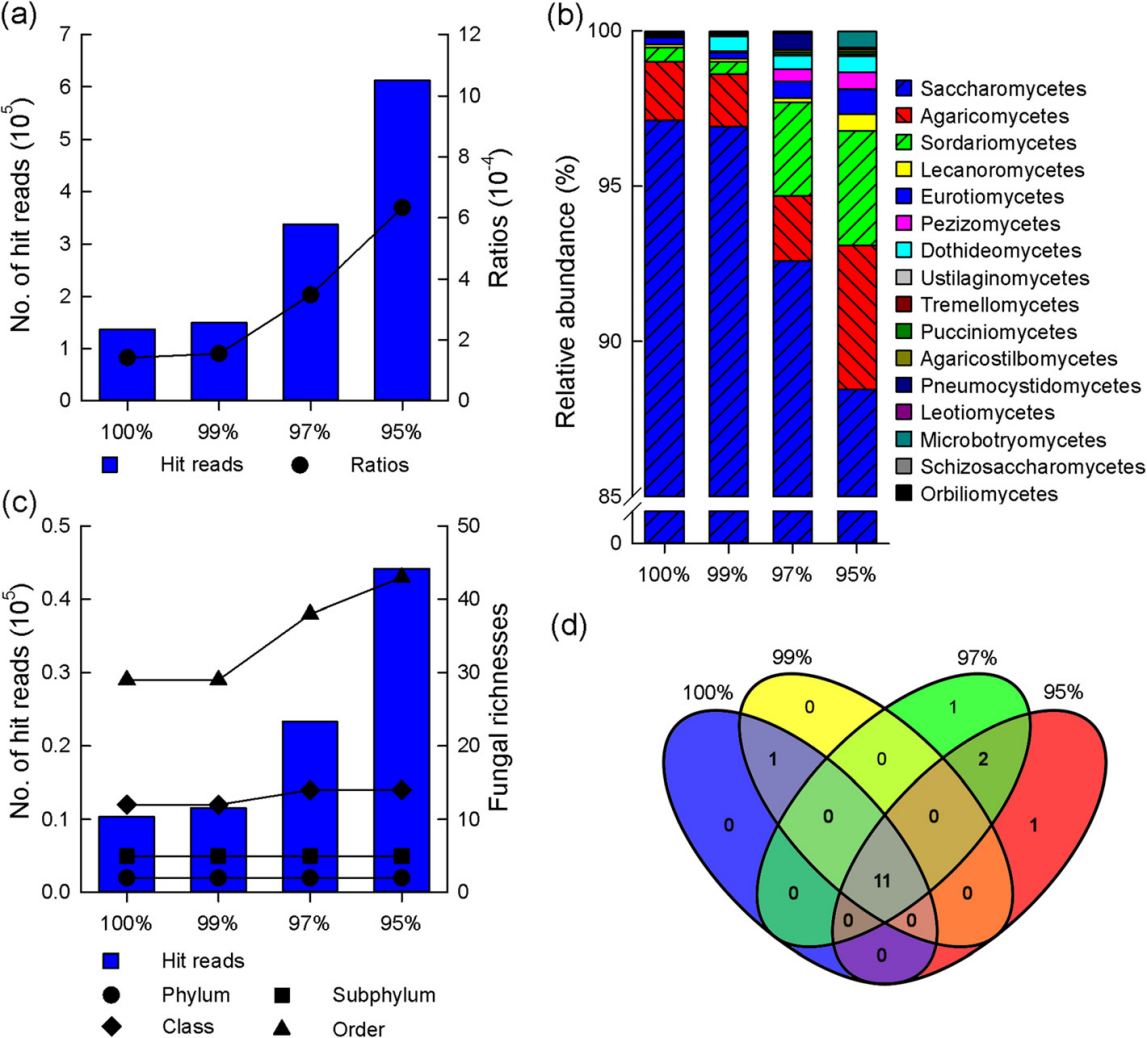
**Fungal ITS data in the unmapped raw data by different thresholds of sequence identity. a**, the number of fungal ITS reads and ratio relative to the unmapped raw data. **b**, the number of fungal ITS reads based on FC1s at the class level and relevant fungal richness at different taxonomic ranks based on FC1s. **c**, relative abundance of the fungal diversity at the class level based on FC1s. **d**, overlap of the fungal communities at the class level based on different minimum threshold of sequence identity.

**Figure 2:**
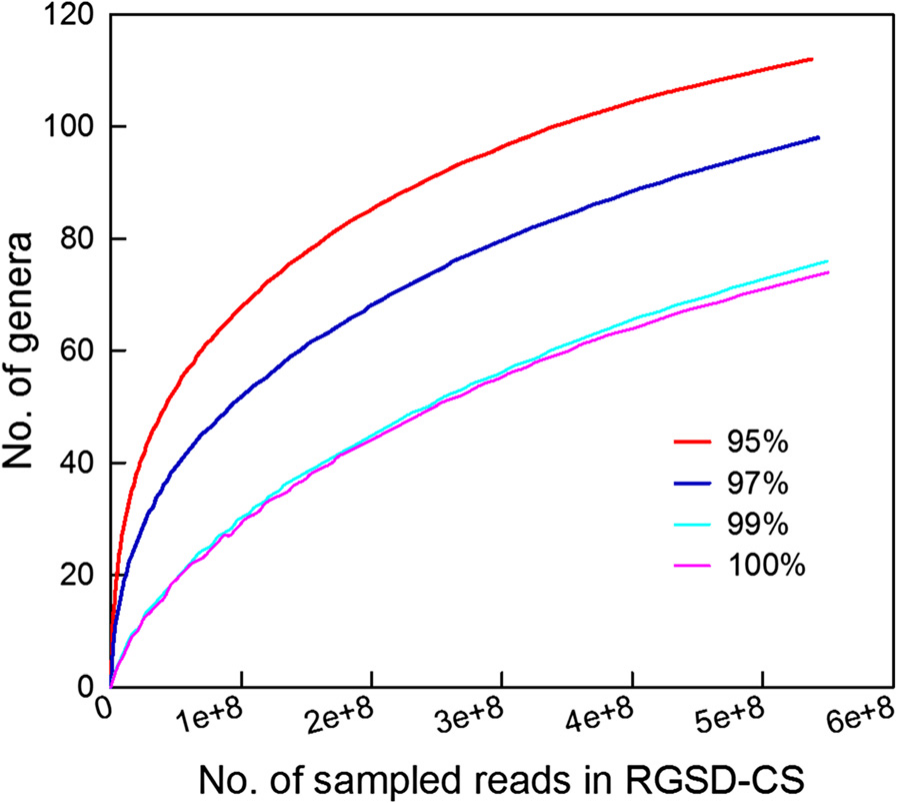
**Rarefaction curves for the richness of fungal community by different thresholds of sequence identity.**

Using bowtie, different fungal communities were determined by pairwise alignments with the thresholds of sequence identities ranging from 100 to 95% between the unmapped raw data and ITS reference database. Base on the four sequence identities of 100%, 99%, 97%, and 95%, the number of matched ITS reads in the unmapped raw data increased from 14,977 to 61,224 (http://www.regulatoryrna.org/pub/figwasp/fungal/), and the ratios of matched ITS reads to the unmapped raw data ranged from 0.00014 to 0.00063. The fungal communities assessed by the four identity thresholds were similar to each other at high taxonomic levels (Figure 1). The fungal communities were composed of 12 to 14 classes and five subphyla, which was revealed by the matched reads in the unmapped raw data, each of which hit just one fungal taxon based on the matched ITS reference sequences (the fungal community based on the reads which just hit one taxon, abbr. FC1s). Nevertheless, the richness of fungal community revealed by 95% identity was about 1.8 times to that of 100% identity at the genus level (Figure 2). These results showed that the richness of fungal community in the unmapped raw data increased as the decline of identity threshold allowed for alignment. It appeared that a smaller identity threshold should be optimal for a thorough investigation of fungal community in the unmapped raw data. Therefore, 95% sequence identity was determined as a suitable threshold, which was used for further investigation of the fungal community composition based on the unmapped raw data in this study [26,27].

### Comparison of fungal diversity based on the ITS and LSU sequence datasets

In this study, two DNA-fragment reference datasets, ITS and LSU of rRNA, were both used to investigate the fungal community based on the unmapped raw data with 95% identity as the threshold for pairwise alignment. In the unmapped raw data, 61,224 raw fungal ITS reads were obtained. However, only 89 raw fungal LSU reads were obtained in the unmapped raw data. The richness of fungal community based on the ITS reference dataset was at least four times more than that based on the LSU reference dataset at the subphylum level. The fungal ITS reads in the unmapped raw data represented at least 14 classes and the raw LSU reads only discovered two classes. At lower taxonomic levels, the unmapped raw data contained only five genera based on LSU but at least 85 families and 158 genera based on ITS. Moreover, all of the fungal taxa that were determined based on LSU sequences were contained in the fungal taxa determined based on ITS sequences. Therefore, as more members of the fungal community were revealed by the ITS dataset than the LSU dataset, the ITS reference dataset was selected as the optimal reference dataset for the investigation of fungal community in this study.

### Fungal community in RGSD-CS

**Figure 3:**
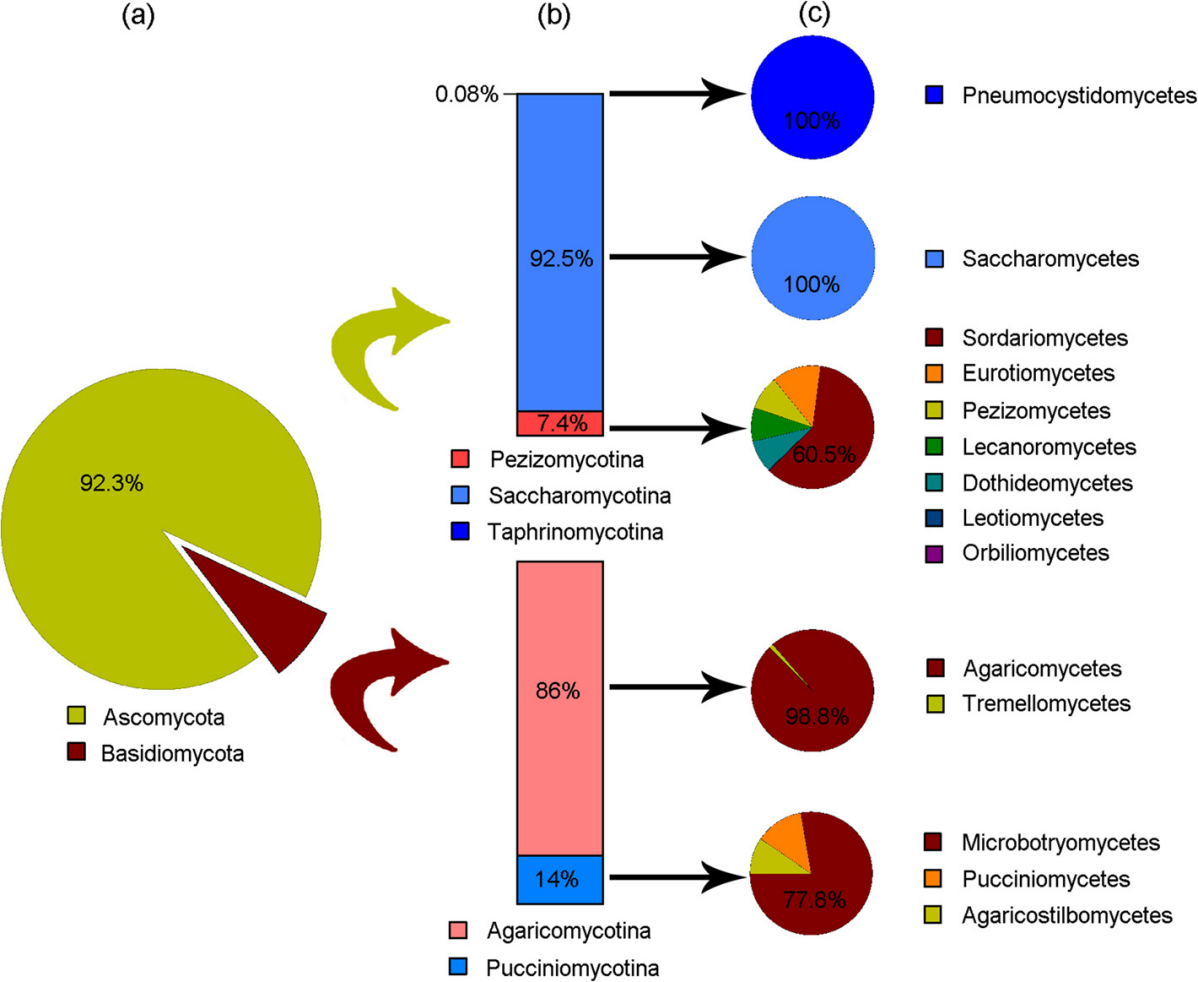
**Fungal community in the unmapped raw data based on FC1 by threshold of 95% identity. a**, fungal community at the phylum level. **b**, fungal community at the subphylum level. **c**, fungal community at the class level.

Based on the fungal community that was represented by the total matched reads in RGSD-CS (fungal community based on the total matched reads, abbr. FCT), up to seven phyla, 28 classes, and 1310 genera were represented in the unmapped raw data by the threshold of 95% sequence identity. Nevertheless, some reads with FCT, each of which simultaneously matched to multiple reference sequences corresponding to different taxa, resulted in the overestimation of the richness of fungal community. Furthermore, exact calculation for the real abundance of these fungal taxa was not possible. Alternatively, FC1 was represented by the reads in RGSD-CS, each of which just matched one taxonomic group and revealed the most conserved fungal community. Based on FC1, all obtained fungi belonged to Dikarya and comprised only two phyla, five subphyla, and 14 classes (Figure 3).

Different from FCT, the relative abundance of each discovered fungal taxon in FC1 was exactly calculated. Saccharomycotina and Pezizomycotina were the most dominant subphyla in the unmapped raw data and in total accounted for 94.5% of the fungal community based on FC1. Agaricomycotina, as the predominant subphylum in Basidiomycota (Figure 3), was the third most dominant subphylum in the unmapped raw data and just accounted for 4.6% of the fungal community based on FC1. As the most abundant class, Saccharomycetes accounted for 88.4% of all fungi in the unmapped raw data based on FC1. Agaricomycetes and Sordariomycetes were the second and third most abundant classes, comprising 4.6% and 3.7% of the fungal community in the unmapped raw data based on FC1, respectively. Following Sordariomycetes, the relative abundances of Eurotiomycetes, Pezizomycetes, Lecanoromycetes, and Microbotryomycetes were similar to each other (averaged 0.5%), and the remaining classes represented about 0.3% of the unmapped raw data together based on FC1 (Additional file 1).

In total, 158 genera were obtained in the unmapped raw data based on FC1 by the threshold of 95% sequence identity. The three most dominant genera belonged to Saccharomycetes and included *Galactomyces*, *Saccharomycopsis*, and *Debaryomyces*. The corresponding raw reads of the three genera in the unmapped raw data ranged from 3308 to 20,531 (Additional file 2), which accounted for 87.9% of the fungal community totally. The three dominant genera of Agaricomycetes were *Inocybe*, *Tricholoma*, and *Lactarius*, which accounted for 2.4%, 0.8%, and 0.8% of the fungal community, respectively. *Ophiocordyceps* and *Trichoderma* were the most dominant genera of Sordariomycetes.

### Comparison of fungal communities in the reported fig-fig wasp symbionts and the unmapped raw data

The fungal ITS sequences from Martinson et al. [25] were composed of 80 and 233 sequences which were obtained from fig wasps and figs, respectively. The 80 fungal ITS sequences of fig wasps belonged to two classes (Saccharomycetes and Dothideomycetes) and three genera (*Metschnikowia*, *Candida*, and *Cladosporium*) (Additional file 3). The remaining 233 fungal ITS sequences of figs represented six fungal classes and 18 genera (Additional file 4). All the three fungal genera of fig wasps were included in the 18 fungal genera of figs.

**Figure 4:**
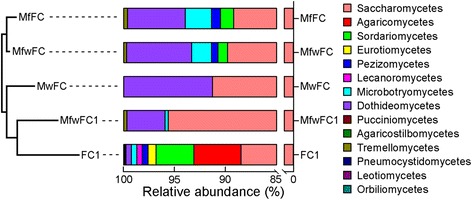
**Fungal community at the class level.** The bar chart on the right represents the fungal composition at the class level. The histogram on the left represents the neighbour-joining clustering of the fungal communities at the class level using Bray-Curtis by past. FC1, indicates the fungal community that corresponds to all of the reads in the unmapped raw data, each of which was matched by only one reference sequence in the ITS dataset. MfwFC1, indicates the fungal community that corresponds to all the reads in the unmapped raw data, each of which was matched by only one reference ITS sequence of figs and wasps from Martinson et al. [25]. MfwFC, indicates the fungal community that corresponds to the ITS sequences of figs and wasps systems from Martinson et al. [25]. MfFC, indicates the fungal community that corresponds to the ITS sequences of figs from Martinson et al. [25]. MwFC, indicates the fungal community that corresponds to the ITS sequences of wasps from Martinson et al. [25].

The abundance of Saccharomycetes, which accounted for 88.4% and 91.3% of fungal communities of figs and fig wasps from Martinson et al. [25], respectively, was similar to that of FC1 (88.4%) in the unmapped raw data. In contrast, Dothideomycetes, the second most abundant class in the previously reported fungal community of figs (5.6%) and fig wasps (8.7%), only represented 0.5% of the fungal community in FC1 (Figure 4). It was noteworthy that Agaricomycetes and Sordariomycetes, the second and third most abundant classes in FC1, were entirely absent in both the previously reported fungal communities of figs and fig wasps.

All of the 18 previously reported fungal genera existed in FCT and partly in FC1. *Metschnikowia*, the most dominant fungal genus in the previously reported fungal community of fig wasps (38.8%, Additional file 3), was represented by only 1918 reads in FCT. In contrast, *Cladosporium* (Dothideomycetes: Capnodiales), the third most dominant fungal genus in the previously reported fungal community of fig wasps (8.7%), was represented by 5586 reads in FCT, which is about 3.3 times more than that of *Metschnikowia*. The abundance of *Candida*, the second dominant genus in the previously reported fungal community of fig wasps (15%), was about four times than that of *Metschnikowia* in the unmapped raw data. It was noteworthy that the three dominant genera in the previously reported fungal community of fig wasps were only presented in FCT, but absent or rare from FC1, which indicated their low abundance in the unmapped raw data. In conclusion, there were significant differences in the fungal communities between the reported fig wasps and the unmapped raw data.

Alignment between the ITS sequences from Martinson et al. [25] and the unmapped raw data using bowtie revealed 899 matched reads. The relevant FC1 and FCT of the previously reported fig-fig wasps represented by these matched reads were compared with the previously reported fungal communities of figs and fig wasps through the neighbour-joining clustering method. Except for the absence of two negligible classes, Microbotryomycetes and Tremellomycetes, the relevant FC1 of the previously reported fig-fig wasps was more similar to the previously reported fungal communities of figs and fig wasps (Figure 4).

### Total size of fungal data estimated in RGSD-CS

In this study, all of the 773 assembled and released fungal genomes were aligned to the unmapped raw data, as determined using bowtie with the threshold of 95% identity. Approximately 0.68 million raw reads were identified and they accounted for nearly 0.71% of the unmapped raw data and 0.12% of RFSD-CS.

In total, 402 ITS sequences corresponding to 402 released fungal genomes, were found in the ITS reference dataset and the remaining ITS sequences of the 371 fungal genomes were not included in the dataset. The 402 ITS sequences and their corresponding fungal genomes were aligned to the unmapped raw data using bowtie with the threshold of 95% identity. Up to 15,206 fungal ITS reads were obtained based on the 402 ITS reference sequences, which is about a quarter of 61,224 fungal ITS reads based on the ITS reference dataset (118,603 reference sequences). The matched raw reads in the unmapped raw data based on the 402 assembled fungal genome accounted for 0.69% of the unmapped raw data, which is close to that based on 773 assembled fungal genomes. These results indicated that the fungal community identified by the 402 ITS reference sequences represented a dominant group of the fungal community determined by the ITS reference dataset.

Subsequently, the ratio of the matched raw reads in the unmapped raw data based on the 402 fungal genomes to those based on their corresponding 402 ITS sequences, was approximately 44.1. This ratio allowed the estimation of the total number of fungal raw reads in the unmapped raw data, which was 2,715,759 (number of total ITS reads (61,224) based on the ITS reference dataset times 44.1). This value accounted for 2.81% and 0.48% of the unmapped raw data and RGSD-CS, respectively.

## Discussion

### The first report of fungal community in RGSD-CS

The raw genomic sequence data of plants and animals were commonly composed of two components, the host genome and the metagenome of microbes. To date, scientists have paid a considerable attention to the analysis of host genomes [28], but not to the microbial metagenome related to those host genomes. Considering the valuable application of raw genomic sequence data, we first explored the fungal community in RGSD-CS.

In this study, up to 17% of the unmapped raw data was achieved in RGSD-CS by excluding the assembled genome of *C. solmsi* which matched RGSD-CS with 100% similarity. The high weight of the unmapped raw data in RGSD-CS supported our speculation that the hundreds of gigabytes of raw genomic sequence data of insects contained a large amount of microbial sequence data in addition to the targeted genomic sequences of insects, and provided an important resource for the subsequent screening of fungal communities.

A suitable threshold of sequence identity for pairwise alignment was important to achieve the correct richness and composition of the fungal community in the unmapped raw data. In this study, the threshold of 95% sequence identity was employed to explore the fungal community in the unmapped raw data. There were two results supporting this selection. First, the matched fungal raw sequences and the richness of the fungal community in the unmapped raw data increased gradually with the decline of the threshold of sequence identity at multiple taxonomic levels. The threshold of 95% sequence identity revealed much more matched fungal reads and fungal richness in the unmapped raw data than that by identities above 95% based on both FC1 and FCT (Figure 1). Second, many studies have demonstrated the feasibility of employing the threshold of 95% sequence identity of ITS genes to discriminate between the intra- and inter- fungal species [26,27]. In contrast, uniform thresholds of sequence identities of intra- and inter- genus, family, and other higher taxonomic levels, were rarely used or defined, thereby making it difficult for us to assign the sequences with matched identity below 95%, into the correct taxa. Therefore, we concluded that the threshold of 95% sequence identity for pairwise alignment, was suitable for investigating the fungal community in the unmapped raw data. However, it was inevitable that some new species and genera that emerged in *C. solmsi* may be overlooked due to the limited sequence identity of 95%.

Both ITS and LSU sequences have previously been used to identify fungal species [29,30]. However, the ITS dataset revealed nearly 30 times the amount of fungal genera more than that revealed by the LSU dataset in the unmapped raw data. This considerable difference of fungal communities between ITS and LSU datasets may be caused by two reasons. First, the low number of LSU reference sequences probably limited the match between raw reads in the unmapped data and the reference dataset. As calculated results, the number of matched reads per LSU reference sequence based on the previous LSU reference dataset (1981 sequences) increased from approximately 0.015 to 0.037 when it was based on the updated LSU reference dataset (2362 sequences). This result indicated that the number of matched reads per LSU reference sequence increased with the increase of the reference dataset. Second, the fungal richness of the two sequence datasets also varied greatly. For example, the ITS dataset contained sequences of 36 classes in Dikarya, but the updated LSU dataset comprised only 27 classes in Dikarya. The lower taxonomic richness of the LSU dataset also limited its scope of application. In this study, the ITS dataset identified more fungal reads than the LSU dataset and was therefore more appropriate as a reference dataset for the further investigation of the fungal community in the raw genomic sequence data.

### The large size of fungal reads in RGSD-CS

These low-abundance or rare microbial groups were often not detected because of limited sequencing depth by traditional methods, including DGGE or clone libraries. In recent years, the use of metabarcoding of fungal DNA (e.g., LSU) by scientists to screen fungal communities of insect hosts has gradually increased, due to its large output of sequence data [14,31]. In this study, at least 0.06 million fungal ITS reads were mined in the unmapped raw data using the threshold of 95% identity for alignment, which was close to the output by metabarcoding of fungal DNA [32]. Furthermore, the number of matched ITS sequences in the unmapped raw data would continuously increase with the decline of the threshold of sequence identity for pairwise alignment (Figure 1). It was noteworthy that the relatively shorter length of raw reads in RGSD-CS, which limited the accurate identification to the subject ITS sequences, would reduce the fungal community revealed by this method. Regardless of the drawback, we estimated that screening RGSD-CS could reveal the richness of fungal community not less than that by high-throughput sequencing based on a single gene. However, the detailed comparison between them was absent here, for the absence of high-throughput sequencing based on the metabarcoding of *C. solmsi*.

The total number of fungal reads in RGSD-CS was estimated by independently aligning the unmapped raw data to the 402 assembled fungal genomes and their corresponding ITS sequences separately. The ratio of matched reads based on the fungal genomes to relevant ITS sequences was approximately 44.1. The considerable number of ITS copies in fungal genomes possibly contribute to the low ratio obtained in this study [33]. Subsequently, the total number of fungal reads in the unmapped raw data was calculated to be 2.7 million based on the total number of matched ITS reads by the threshold of 95% sequence identity. However, the number of total fungal reads in RGSD-CS was underestimated in this study, because of the relatively high threshold of 95% sequence identity. Therefore, the percentage of total fungal reads were estimated to be at least 0.48% in RGSD-CS. Anyway, this result supported the view that RGSD-CS contained large amounts of fungal sequences.

In addition, the remaining data of the unmapped raw data still accounted for about 16.57% of RGSD-CS, which was remarkably more than that accounted by the fungal community. Moreover, the remaining unmapped raw data was still a rich resource to explore other microbial information contained in RGSD-CS, such as bacteria and virus. Unfortunately, the total number of fungal reads in RGSD-CS was significantly smaller than the size of a metagenome [34], rendering it difficult to assemble the metagenome of *C. solmsi*.

### The fungal community in RGSD-CS

This study was the first to systematically detect the fungal community of male fig wasps based on RGSD-CS. In this study, disregarding some new fungal species and genera that might be overlooked due to the conserved sequence identity threshold of 95%, there were still nearly 158 genera and 14 classes that were determined in the unmapped raw data based on FC1. It was likely that the fungal community revealed in the unmapped raw data was the richest compared with those of other reported insects that were assessed by traditional [5,6,14,35] and metabarcoding methods [14]. Although the dominant classes (Saccharomycetes, Agaricomycetes, Sordariomycetes, Eurotiomycetes, and Pezizomycetes) and genera (*Galactomyces*, *Saccharomycopsis*, *Debaryomyces*, *Inocybe*, *Tricholoma*, *Lactarius*, *Ophiocordyceps*, and *Trichoderma*) in the unmapped raw data also commonly existed in other insects as well as in fig wasps, they did not coexist in other insects that were screened by other culture-dependent and -free methods [6,24,25,35-46]. This might indicate that the considerable amount of fungal data in RGSD-CS could help discover the low-abundance and rare fungi that were ignored by the small amount of fungal data in other studies.

Although the amount of identified raw reads promised a good representation of the real fungal community, it must be noted that the rarefaction curves based on all the four sequence identities did not approach the plateau as the 567,430,494 reads of RGSD-CS corresponding to 92.95× sequencing coverage (Figure 2). There were still many unknown fungal genera associated with *C. solmsi,* which were not discovered in this study. This result suggested that a deeper sequencing coverage and more unmapped raw data were required to obtain the entire fungal community of *C. solmsi*.

The main components of FC1 and the previously reported fungal community of fig wasps in the unmapped raw data were similar to each other (Figure 4). For example, at the class level, Saccharomycetes was the most abundant fungal class of *C. solmsi* in both FC1 and the previously reported fungal community of fig wasps; at the genus level, the most dominant genus in the previously reported fungal community of fig wasps, *Candida*, was also an important component in FC1. This finding suggested that the fungal information mined from the raw genomic sequence data could be used to confirm the fungal community in fig wasps, as discovered by traditional methods [25]. However, some significant differences still exist. Agaricomycetes and Sordariomycetes, which were absent in all of the other six fig wasp species [25], were the second and third most abundant classes in RGSD-CS and may be unique to *C. solmsi*. Moreover, some other classes in *C. solmsi*, such as Lecanoromycetes, Eurotiomycetes, and Exobasidiomycetes, were also absent in the six other fig wasp species. In contrast, Dothideomycetes, the second most abundant class in other fig wasps, was relatively rare in *C. solmsi*.

There were several possible reasons for these differences on the richness and composition of fungal community. First, the enormous difference in sequence number should be the most important factor contributing to the difference in richness of fungal community. Compared with the 313 cloned sequences from Martinson et al. [25], the matched 61,224 reads in the unmapped raw data by the threshold of 95% sequence identity mined dramatically more richness of fungal community, particularly those low abundant components. Second, host taxa might play an important role on the composition of fungal communities [47]. *C. solmsi* (Agaonidae; Agaoninae) has been separated from the other six distantly related fig wasp species (Chalcidoidea; Agaonidae; Agaoninae; *Pegoscapus* and *Tetrapus*) for 50–90 million years [48]. The significant genetic divergence of *C. solmsi* from the six other fig wasp species impeded the interaction among the species [48]. Third, the extreme sexual dimorphism and functional differences of male and female wasps might affect fungal community composition. The samples of RGSD-CS were all male, but the fig wasps studied in the other studies were all female. However, no study to date has compared the fungal communities between male and female insects. Fourth, 500 individuals of *C. solmsi* were pooled together for the DNA extraction. The large sample size was much larger than that has been used for most other insects [6] and significantly reduced biases among fungal communities of individuals. Finally, compared with the wasps in Martinson et al. [25] which were surface sterilized with 70% ethanol, the wasps in this study was simply washed with double-distilled water. Then the surface microbes of wasps might contribute to some taxa assessed in RGSD-CS.

### Potential relationship between *C. solmsi* and related fungi

The mutualism between *Ficus hispida* and its pollinating wasp (*C. solmsi*) has coevolved for nearly 90 million years [49,50]. The male *C. solmsi* has undergone considerable morphological and genomic evolution to adapt to the extreme environment in the closed syconium. Because of the negligible attention to the fig wasp fungal community as well as the difficulty associated with culturing fig wasps in the lab, the fungal community of and its interaction with male wasps was unclear.

The presence of Saccharomycetes might typically reflect the feeding types of the host [49]. The most dominant genera in RGSD-CS, *Galactomyces*, *Saccharomyces*, *Debaryomyces, Pichia*, and *Candida*, all belonged to Saccharomycetes and were widely distributed in many phytophagous insects [38,46], as well as in other fig wasp species [25]. Many strains isolated from *Galactomyces* and *Debaryomyces* could efficiently degrade plant cell wall polysaccharides, which might add in the digestion of polysaccharides for insect hosts [51]. Many species of *Saccharomyces* and *Candida* took part in the degradation of substrates by secreting amylases, acid protease, and β-glucosidase, which contribute to the synthesis of essential nutrient elements in insect hosts [52]. Herein, we hypothesized that these dominant fungi might play roles in the development of *C. solmsi*, which must uptake various types of externally obtained essential nutrients, such as histidine, isoleucine, leucine, lysine, methionine, phenylalanine, threonine, and tryptophan [22]. Additionally, some species of *Saccharomyces* and *Candida* were also reported to contribute to the ergosterol biosynthetic pathway in many insects [53,54]. Moreover, although absent from the six other reported fig wasps, Agaricomycetes was the second most dominant class of *C. solmsi*. However, little was known regarding the role of Agaricomycetes in the insect hosts [55,56]. Ultimately, some specific relationships between fungi and their wasp hosts remain unclear, as described above.

## Conclusions

In this study, we first investigated the fungal community in RGSD-CS based on the ITS reference dataset and estimated the size of fungal data in RGSD-CS. This study provided new insights into the fungal community from the raw genomic sequence data of hosts as well as a basic procedure to efficiently analyse microbial diversity using raw genomic sequence data. Additionally, functions of these fungal communities will be further explored by detailed investigation of the functional genes of fungi in the raw genomic sequence data. Other research can also be carried out by biologists using this method to examine the raw genomic sequence data of other animal or plant hosts.

## Methods

### Raw genomic sequence data of *C. solmsi*

RGSD-CS was used to investigate the fungal information that was related to *C. solmsi*. About five hundred male fig wasps were collected from naturally matured figs in 2010 and immediately stored in liquid nitrogen after thoroughly washing with double-distilled water [22]. The total DNA of the 500 pooled individuals of *C. solmsi* was extracted using a method that was modified from the protocol developed by J. Rehm for the Berkeley *Drosophila* Genome Project [57].

The genome of *C. solmsi* was sequenced to 92.95× average coverage using the Illumina-HiSeq™ 2000 platform with the paired-end sequencing approach. Approximately 44.63 Gbp data were obtained, and 12.32 Gbp of high-quality sequences were used for the *C. solmsi* genome assembly (294 Mbp). The high-quality data, which contained 567,430,494 raw reads [22], were defined as RGSD-CS for the analysis of fungal information.

### The reference datasets

Two DNA sequence datasets of fungi, the BLAST and SILVA datasets, were selected as the reference datasets to help determine fungal community. The BLAST dataset (http://www.emerencia.org/fungalitspipeline.html) contained 118603 fungal ITS sequences that consisted of 10 phyla, 11 subphyla, and 36 classes. The SILVA dataset contained 2362 fungal LSU sequences that consisted of 10 phyla, 10 subphyla, and 27 classes (http://www.arb-silva.de/).

All of the 773 released fungal genomes (updated Sept 2013) were downloaded from NCBI (ftp://ftp.ncbi.nlm.nih.gov/refseq/release/fungi/) and used as reference genomes. According to our statistics, these genomes were distributed in more than 10 phyla, seven subphyla, 27 classes, and 773 species.

A total of 313 fungal ITS sequences that were related to the fig-fig wasp system and described by Martinson [25] were also downloaded from NCBI. We classified the 313 fungal ITS sequences of fig-fig wasp systems into six classes, eight orders, 18 genera, and 24 species by local blast using the fungal ITS reference dataset.

### Analytical procedure

All statistics in this study was conducted by amounts of shell and python scripts, which were detailed in Additional files 5 and 6.

1. The unmapped raw data of RGSD-CS was obtained by excluding the completely assembled genomic sequence of *Ceratosolen solmsi* (accession no. ATAC01000000) using bowtie. The unmapped raw data (http://www.regulatoryrna.org/pub/figwasp/fungal/) which was predominantly accounted by microbial sequences, including fungal, bacterial and viral sequences was employed for the following analysis.
2. The unmapped raw data was aligned to the two reference sequence datasets that were described above using bowtie with parameters --best and –strata, which aid in hitting and reporting guaranteed best stratum. The matched sequences in the reference datasets and corresponding taxonomic diversity of fungi were summarized through a series of shell scripts. The taxonomy of matched ITS reference sequences in the BLAST dataset were further verified with manually cured dataset UNITE [58] by local blastn. The statistics of abundance of each fungal taxon that corresponded to the matched raw reads was also carried out. The two reference datasets were compared by the richness of fungal community, and the better dataset was selected for the following steps.
3. In order to assess the feasibility of this approach, RGSD-CS was also aligned to the sequences from Martinson et al. [25] using bowtie. Based on the fungal community compositions at class level, a clustering tree was generated by past with Bray-Curtis measure to display their similarity.
4. In order to estimate the proportion of fungal sequences within the unmapped raw data, the unmapped raw data was aligned to the 773 released fungal genomes. Further, a portion of the representative ITS sequences of the 773 genomes were collected and also aligned to the unmapped raw data. The matched sequences from these fungal genomes, representative ITS sequences, and corresponding taxonomic diversity of fungi were summarized. The proportion of total fungal reads in the unmapped raw data was calculated.
5. In order to assess the relationship between the richness of fungal community in the unmapped raw data and the number of raw reads in RGSD-CS, the rarefaction measure was performed using a python script.

## Availability of supporting data

The raw data of fungal ITS gene in *C. solmsi* genome supporting the results of this article are available in LabArchives repository, doi: 10.6070/H46T0JNQ (http://ezid.cdlib.org/id/doi:10.6070/H46T0JNQ) and doi:10.6070/H4319SW9 (http://ezid.cdlib.org/id/doi:10.6070/H4319SW9). The other data supporting the results of this article are include within the article and its additional files.

## Additional files

Additional file 1:
**Fungal community at the class level based on the threshold of 95% identity.**
Additional file 2:
**The first 20 dominant genera in RGSD-CS based on the threshold of 95% identity.**
Additional file 3:
**Fungal taxonomy related to the 80 fungal ITS sequences of six other fig wasp species.**
Additional file 4:
**Fungal taxonomy related to the 233 fungal ITS sequences of six other fig species.**
Additional file 5:
**Shell scripts applied in this study.**
Additional file 6:
**Python scripts applied in this study.**

## Abbreviations

RGSD-CS: the raw genomic sequence data of *Ceratosolen solmsi*
ITS: internal transcribed spacer of rRNA
LSU: large ribosomal subunit of rRNA
FCT: the fungal community represented by the total matched reads in RGSD-CS based on the ITS reference dataset.
FC1: the fungal community represented by the matched reads in RGSD-CS, each of which was matched by only one fungal taxonomic group based on the matched reference sequence in the ITS reference dataset
