# Supplementary material for: New insights into the fungal community from the raw genomic sequence data of fig wasp Ceratosolen solmsi
Source: BMC Microbiol. 2015 Feb 12;15(1):27. doi: 10.1186/s12866-015-0370-3 (PMC4329198; doi:10.1186/s12866-015-0370-3)
Supplement: Additional file 1: — Fungal community at the class level based on the threshold of 95% identity. [file 12866_2015_370_MOESM1_ESM.pdf]

Additional file 1. Fungal community at the class level based on the threshold of 95% identity.

Class	Percentage (%)	No. of hit reads
Saccharomycetes	88.459	39068
Agaricomycetes	4.635	2047
Sordariomycetes	3.702	1635
Eurotiomycetes	0.788	348
Pezizomycetes	0.550	243
Lecanoromycetes	0.543	240
Microbotryomycetes	0.539	238
Dothideomycetes	0.509	225
Pucciniomycetes	0.088	39
Agaricostilbomycetes	0.066	29
Tremellomycetes	0.054	24
Pneumocystidomycetes	0.050	22
Leotiomycetes	0.014	6
Orbiliomycetes	0.002	1
