# Supplementary material for: New insights into the fungal community from the raw genomic sequence data of fig wasp Ceratosolen solmsi
Source: BMC Microbiol. 2015 Feb 12;15(1):27. doi: 10.1186/s12866-015-0370-3 (PMC4329198; doi:10.1186/s12866-015-0370-3)
Supplement: Additional file 2: — The first 20 dominant genera in RGSD-CS based on the threshold of 95% identity. [file 12866_2015_370_MOESM2_ESM.pdf]

Additional file 2. The first 20 dominant genera in RGSD-CS based on the threshold of 95% identity.

Phylum	Subphylum	Class	Order	Family	Genus	No. of hit reads
Ascomycota	Saccharomycotina	Saccharomycetes	Saccharomycetales	Dipodascaceae	*Galactomyces*	20531
Ascomycota	Saccharomycotina	Saccharomycetes	Saccharomycetales	Saccharomycopsidaceae	*Saccharomycopsis*	9215
Ascomycota	Saccharomycotina	Saccharomycetes	Saccharomycetales	Debaryomycetaceae	*Debaryomyces*	3308
Basidiomycota	Agaricomycotina	Agaricomycetes	Agaricales	Inocybaceae	*Inocybe*	890
Ascomycota	Pezizomycotina	Sordariomycetes	Hypocreales	Ophiocordycipitaceae	*Ophiocordyceps*	628
Basidiomycota	Agaricomycotina	Agaricomycetes	Agaricales	Tricholomataceae	*Tricholoma*	321
Basidiomycota	Agaricomycotina	Agaricomycetes	Russulales	Russulaceae	*Lactarius*	318
Ascomycota	Pezizomycotina	Sordariomycetes	Hypocreales	Hypocreaceae	*Trichoderma*	227
Basidiomycota	--	Urediniomycetes	Sporidiales	incert sedis	*Rhodotorula*	224
Ascomycota	Pezizomycotina	Eurotiomycetes	Onygenales	Onygenaceae	*Renispora*	200
Ascomycota	Pezizomycotina	Lecanoromycetes	Peltigerales	Peltigeraceae	*Peltigera*	158
Ascomycota	Pezizomycotina	Pezizomycetes	Pezizales	Rhizinaceae	*Phymatotrichopsis*	126
Basidiomycota	Agaricomycotina	Agaricomycetes	Sebacinales	Sebacinaceae	*Sebacina*	123
Ascomycota	Pezizomycotina	Pezizomycetes	Pezizales	Ascobolaceae	*Ascobolus*	109
Ascomycota	Pezizomycotina	Dothideomycetes	Pleosporales	Pleosporaceae	*Alternaria*	108
Ascomycota	Saccharomycotina	Saccharomycetes	Saccharomycetales	Saccharomycetaceae	*Pichia*	104
Ascomycota	Pezizomycotina	Sordariomycetes	Hypocreales	Clavicipitaceae	*Metarhizium*	86
Ascomycota	Pezizomycotina	Eurotiomycetes	Onygenales	Arthrodermataceae	*Trichophyton*	79
Ascomycota	Pezizomycotina	Dothideomycetes	Botryosphaeriales	Botryosphaeriaceae	*Guignardia*	61
Basidiomycota	Agaricomycotina	Agaricomycetes	Agaricales	Hygrophoraceae	*Dictyonema*	56
