# Supplementary material for: New insights into the fungal community from the raw genomic sequence data of fig wasp Ceratosolen solmsi
Source: BMC Microbiol. 2015 Feb 12;15(1):27. doi: 10.1186/s12866-015-0370-3 (PMC4329198; doi:10.1186/s12866-015-0370-3)
Supplement: Additional file 3: — Fungal taxonomy related to the 80 fungal ITS sequences of six other fig wasp species. [file 12866_2015_370_MOESM3_ESM.pdf]

Additional file 3. Fungal taxonomy related to the 80 fungal ITS sequences of six other fig wasp species.

Phylum	Class	Order	Family	Genus	No. of sequences
Ascomycota	Saccharomycetes	Saccharomycetales	Metschnikowiaceae	*Metschnikowia*	31
Ascomycota	Saccharomycetes	Saccharomycetales	Saccharomycetaceae	*Candida*	12
Ascomycota	Saccharomycetes	Saccharomycetales	Saccharomycetaceae	*Saccharomyces*	30
Ascomycota	Dothideomycetes	Capnodiales	Cladosporiaceae	*Cladosporium*	7
