# Supplementary material for: New insights into the fungal community from the raw genomic sequence data of fig wasp Ceratosolen solmsi
Source: BMC Microbiol. 2015 Feb 12;15(1):27. doi: 10.1186/s12866-015-0370-3 (PMC4329198; doi:10.1186/s12866-015-0370-3)
Supplement: Additional file 4: — Fungal taxonomy related to the 233 fungal ITS sequences of six other fig species. [file 12866_2015_370_MOESM4_ESM.pdf]

Additional file 4. Fungal taxonomy related to the 233 fungal ITS sequences of six other fig species.

Phylum	Subphylum	Class	Order	Family	Genus
Ascomycota	Pezizomycotina	Dothideomycetes	Capnodiales	Cladosporiaceae	*Cladosporium*
Ascomycota	Pezizomycotina	Dothideomycetes	Dothideales	Dothideaceae	*Coniozyma*
Ascomycota	Pezizomycotina	Dothideomycetes	Pleosporales	Incertae sedis	*Stagonosporopsis*
Ascomycota	Pezizomycotina	Dothideomycetes	Pleosporales	Phaeosphaeriaceae	*Phaeosphaeriopsis*
Ascomycota	Pezizomycotina	Dothideomycetes	Pleosporales	Pleosporineae	*Alternaria*
Ascomycota	Pezizomycotina	Dothideomycetes	Pleosporales	Pleosporineae	*Curvularia*
Ascomycota	Pezizomycotina	Dothideomycetes	Pleosporomycetidae	Gloniaceae	*Glonium*
Ascomycota	Pezizomycotina	Pezizomycetes	Pezizales	Incertae sedis	*Phialea*
Ascomycota	Pezizomycotina	Sordariomycetes	Hypocreales	Nectriaceae	*Fusarium*
Ascomycota	Pezizomycotina	Sordariomycetes	Xylariales	Diatrypaceae	*Diatrypella*
Ascomycota	Saccharomycotina	Saccharomycetes	Saccharomycetales	Debaryomycetaceae	*Schwanniomyces*
Ascomycota	Saccharomycotina	Saccharomycetes	Saccharomycetales	Metschnikowiaceae	*Clavispora*
Ascomycota	Saccharomycotina	Saccharomycetes	Saccharomycetales	Metschnikowiaceae	*Metschnikowia*
Ascomycota	Saccharomycotina	Saccharomycetes	Saccharomycetales	Phaffomycetaceae	*Wickerhamomyces*
Ascomycota	Saccharomycotina	Saccharomycetes	Saccharomycetales	Saccharomycetaceae	*Candida*
Ascomycota	Saccharomycotina	Saccharomycetes	Saccharomycetales	Saccharomycetaceae	*Saccharomyces*
Basidiomycota	Agaricomycotina	Tremellomycetes	Tremellales	Tremellaceae	*Cryptococcus*
Basidiomycota	Pucciniomycotina	Microbotryomycetes	Sporidiobolales	Incertae sedis	*Rhodotorula*
