# Supplementary material for: New insights into the fungal community from the raw genomic sequence data of fig wasp Ceratosolen solmsi
Source: BMC Microbiol. 2015 Feb 12;15(1):27. doi: 10.1186/s12866-015-0370-3 (PMC4329198; doi:10.1186/s12866-015-0370-3)
Supplement: Additional file 5: — Shell scripts applied in this study. [file 12866_2015_370_MOESM5_ESM.pdf]

Additional file 5. Shell scripts applied in this study.

1: obtain the unmapped raw data

Query data: raw genome sequence data of of *Ceratosolen solmsi*
Object data: assembled genome of *Ceratosolen solmsi*
Script:

```
Bowtie -a –v 0 --best --strata   querydata   --un   objectdata   unmappedrawdata
```

2：obtain the matched fungal sequences in the unmapped raw data with identifies from 93% to 100% seperately. The mismatched base pairs have been calculated according the length of raw sequences.

Query data: the unmapped raw data
Object data: reference database of fungal ITS (or LSU，fungal genome *et al.*) sequences
Script:

```
bowtie -a -v (0-3) --best --strata querydata objectdata –al result2
bowtie -a -n 3 -e 300 -l 60 --best --strata querydata objectdata --al result2
bowtie -a -n 3 -e 300 -l 40 --best --strata querydata objectdata --al result2
```

3：calculate the number of matched reads

Script:

```
cut -f 1 result2 |sort -u |wc -l > result3
```

4: calculate the richness of genera

Script:

```
cut -f 2 result2 |cut -d_ -f 2 |sort -u |wc -l > n_genus
```

5: calculate the richness of species

Script:

```
cut -f 2 result2 | cut -d_ -f 2,3|sort -u |wc -l >n_species
```

Statistics of the total abundance of fungal community

6: to obtain the ID of matched ITS squences in NCBI

Script:

```
cat result2 | awk 'gsub("      ", "_"){print}'|cut -d_ -f 1,2,3| cut -d_ -f 2,3 |sort -t_ -k 2 –u|cut -d_ -f 1 | > ABUNT_id
```

7: download id_taxa

batch entrez （ABUNT_id）

8: id_taxa

Script:

```
cat sequence.gb |
awk -v RS="" '{gsub("\n", "#"); print}'|
awk 'gsub("VERSION", "@VERSION"){print}'|
awk 'gsub("ACCESSION", "ACCESSION@"){print}'|
awk 'gsub("Eukaryota", "@ Eukaryota"){print}'|
awk 'gsub("#REFERENCE", "@#REFERENCE"){print}'|
cut -d@ -f 2,4 |
```

awk 'gsub("#", ""){print}'|
awk 'gsub(" ", ""){print}' |
awk 'gsub("@", " "){print}'|sort > ABUNT_id_taxa

9: Determine taxonomy by UNITE dataset
Query data: matched ITS sequences in BLAST dataset
Object data: ITS sequences in UNITE dataset
Script: blastall -p blastn -d query data -i objectdata -o result -m 9 -e 0.001

10: id_genus
Script:
cat result2 | awk 'gsub("   ", "_"){print}'|cut -d_ -f 1,2,3| cut -d_ -f 2,3 |sort -t_ -k 2 –u|awk 'gsub("_", " "){print}'|sort >ABUNT_id_genus

11: id_genus_taxa
Script:
join ABUNT_id_genus ABUNT_id_taxa >ABUNT_id_genus_taxa

Join Reads and taxa
12: genus_taxa
Script:
cat ABUNT_id_genus_taxa | cut -d" " -f 2,3|sort > abunt_genus_taxa

13： reads_genus
Script:
cat result2 | awk 'gsub("   ", "_"){print}'|cut -d_ -f 1,2,3 |cut -d_ -f 1,3 | awk 'gsub("_", " "){print}'| sort –t" " -k 2 >abunt_read_genus

14： genus_reads_taxa
Script:
Join -1 2 -2 1 abunt_read_genus abunt_genus_taxa > **abunt_genus_read_taxa**

15： abunt_genus
Script:
cat result2 | awk 'gsub("   ", "_"){print}'|cut -d_ -f 1,2,3 |cut -d_ -f 1,3 |sort –u|cut -d_ -f 2 |sort|uniq –c > abunt_genus

16： read_taxa
Script:
cut –d" " -f 2,3 abunt_genus_read_taxa| awk 'gsub("   ", "_"){print}'|awk 'gsub(";", "_"){print}' >abunt_read_taxa

17： abunt_Dikary :
Script:

cut -d_ -f 1,4 abunt_read_taxa |sort -u |cut -d_ -f 2|sort |uniq -c > abunt_Dikary

18: read_taxa_noD
PERL-ITS_noD.pl
:::: abunt_read_taxa >**abunt_read_taxa _noD**

19: abunt_noD_phylum
Script:
cut -d_ -f 1,4 abunt_read_taxa_noD|sort -u |cut -d_ -f 2|sort |uniq -c > abunt_noD_phylum

20: abunt_noD_subphylum
Script:
cut -d_ -f 1,5 abunt_read_taxa_noD|sort -u|cut -d_ -f 2|sort |uniq -c > abunt_noD_subphylum

Statistics of the abundance of fungal community assigned to Dikary
21: read_taxa_onD
Script:
cat abunt_read_taxa |awk '/Dikarya/ {print}' >**abunt_read_taxa_onD**

22: abunt_onD_phylum
Script:
cut -d_ -f 1,5 abunt_read_taxa_onD |sort -u|cut -d_ -f 2|sort |uniq -c > abunt_onD_phylum

23: abunt_onD_subphylum
Script:
cut -d_ -f 1,6 abunt_read_taxa_onD |sort -u|cut -d_ -f 2|sort |uniq -c > abunt_onD_subphylum

24: abunt_onD_class
Script:
cut -d_ -f 1,7 abunt_read_taxa_onD |sort -u|cut -d_ -f 2|sort |uniq -c > abunt_onD_class

Statistics of the abundance of fungal community assigned to Dikary based on the reads each of which only hits one taxa at category levels. (for example: at class level)
25: read_class
Script:
cut -d_ -f 1,7 abunt_read_taxa_onD |sort -u|awk 'gsub("_", " "){print}' >abunt_read_class_onD

26: nr1_read
Script:
cut -d" " -f 1 abunt_read_class_onD|uniq -c |sort –n| awk '{FS=" "}{if($1==1) print$0}'|sort > abunt_read_class_onD_nr

27: nr1_read_class
Script:

join -1 2 -2 1 abunt_read_class_onD_nr abunt_read_class_onD > abunt_read_class_onD_nr1_read_class

28: abunt_nr1_read_class

Script:

cut -d" " -f 3 abunt_read_class_onD_nr1_read_class| sort| uniq -c > abunt_read_class_onD_nr1_abunt_class
