# Supplementary material for: New insights into the fungal community from the raw genomic sequence data of fig wasp Ceratosolen solmsi
Source: BMC Microbiol. 2015 Feb 12;15(1):27. doi: 10.1186/s12866-015-0370-3 (PMC4329198; doi:10.1186/s12866-015-0370-3)
Supplement: Additional file 6: — Python scripts applied in this study. [file 12866_2015_370_MOESM6_ESM.pdf]

Additional file 6. Python scripts applied in this study.

```
#!/usr/bin/env python
import sys
import random

N = 1000
Intv = 100
#sam=pysam.Samfile(sys.argv[1],'rb')
fin = open(sys.argv[1],'r')
out=open(sys.argv[2],'w')

rds = []
rdsps = {}
rdspn = {}
i = 0
last = ""
for l in fin :
    lst = l.strip().split(" ")
    #print lst[0]
    readid = lst[1]
    spinfo = lst[0]
    if(readid != last):
        i += 1; ##
        last = readid
        rds.append(readid)
        rdsps[readid] = [spinfo]
    else:
        if spinfo not in rdsps[readid]:
            rdsps[readid].append(spinfo)

print i, "reads."
rflen = i / Intv + 1
rf = []
rfr = []
for j in range(rflen):
    rf.append([0] * N)
    rfr.append(j * Intv)
rfr[rflen - 1] = i
for rid in rdsps:
    rdspn[rid] = len(rdsps[rid])
    #print rdsps[rid],rdspn[rid],len(rdsps)
#print
rdsps['FCB060YABXX:3:1102:8881:140229#CGATGTAT/1'],rdspn['FCB060YABX
```

```
X:3:1102:8881:140229#CGATGTAT/1']
for n in range(N):
	if n % 10 == 0: print n
	random.shuffle(rds)
	sps = {}
	i = 0
	for rid in rds:
		if i % Intv == 0:
			rf[i / Intv][n] = len(sps)
		i += 1
		if rdspn[rid] == 1 :
			#if rdsps[rid][0] == 'Fusarium':
				#print rid
			if rdsps[rid][0] not in sps:
				sps[rdsps[rid][0]] = 1
			else:
				sps[rdsps[rid][0]] += 1
	rf[rflen - 1][n] = len(sps)

#print sps
out.write("Pos\tmedian\tL95\tU95\n")
for j in range(rflen):
	rf[j].sort()
	mm = (rf[j][N/2]+rf[j][N/2+1])/2
	ll = (rf[j][N/40]+rf[j][N/40+1])/2
	uu = (rf[j][N*39/40]+rf[j][N*39/40+1])/2
	out.write(str(rfr[j]) + "\t" +str(mm) + "\t" +str(ll) + "\t" +str(uu) + "\n")
#	out.write(str(rfr[j]) + "\t" + "\t".join(map(str, rf[j])) + "\n")
```
